# A comprehensive swarming intelligent method for optimizing deep learning-based object detection by unmanned ground vehicles

**DOI:** 10.1371/journal.pone.0251339

**Published:** 2021-05-13

**Authors:** Qian Xu, Gang Wang, Ying Li, Ling Shi, Yaxin Li

**Affiliations:** 1 College of Computer Science and Technology, Jilin University, Changchun, People’s Republic of China; 2 Key Laboratory of Symbolic Computation and Knowledge Engineering of Ministry of Education, Jilin University, Changchun, People’s Republic of China; 3 State Key Laboratory of Automotive Simulation and Control, Jilin University, Changchun, People’s Republic of China; 4 Department of Electronic and Computer Engineering, Hong Kong University of Science and Technology, Clear Water Bay, Kowloon, Hong Kong; 5 College of Automotive Engineering, Jilin University, Changchun, People’s Republic of China; Fuzhou University, CHINA

## Abstract

Unmanned ground vehicles (UGVs) are an important research application of artificial intelligence. In particular, the deep learning-based object detection method is widely used in UGV-based environmental perception. Good experimental results are achieved by the deep learning-based object detection method Faster region-based convolutional neural network (Faster R-CNN). However, the exploration space of the region proposal network (RPN) is restricted by its expression. In our paper, a boosted RPN (BRPN) with three improvements is developed to solve this problem. First, a novel enhanced pooling network is designed in this paper. Therefore, the BRPN can adapt to objects with different shapes. Second, the expression of BRPN loss function is improved to learn the negative samples. Furthermore, the grey wolf optimizer (GWO) is used to optimize the parameters of the improved BRPN loss function. Thereafter, the performance of the BRPN loss function is promoted. Third, a novel GA-SVM classifier is applied to strengthen the classification capacity. The PASCAL VOC 2007, VOC 2012 and KITTI datasets are used to test the BRPN. Consequently, excellent experimental results are obtained by our deep learning-based object detection method.

## Introduction

**Fig 1:**
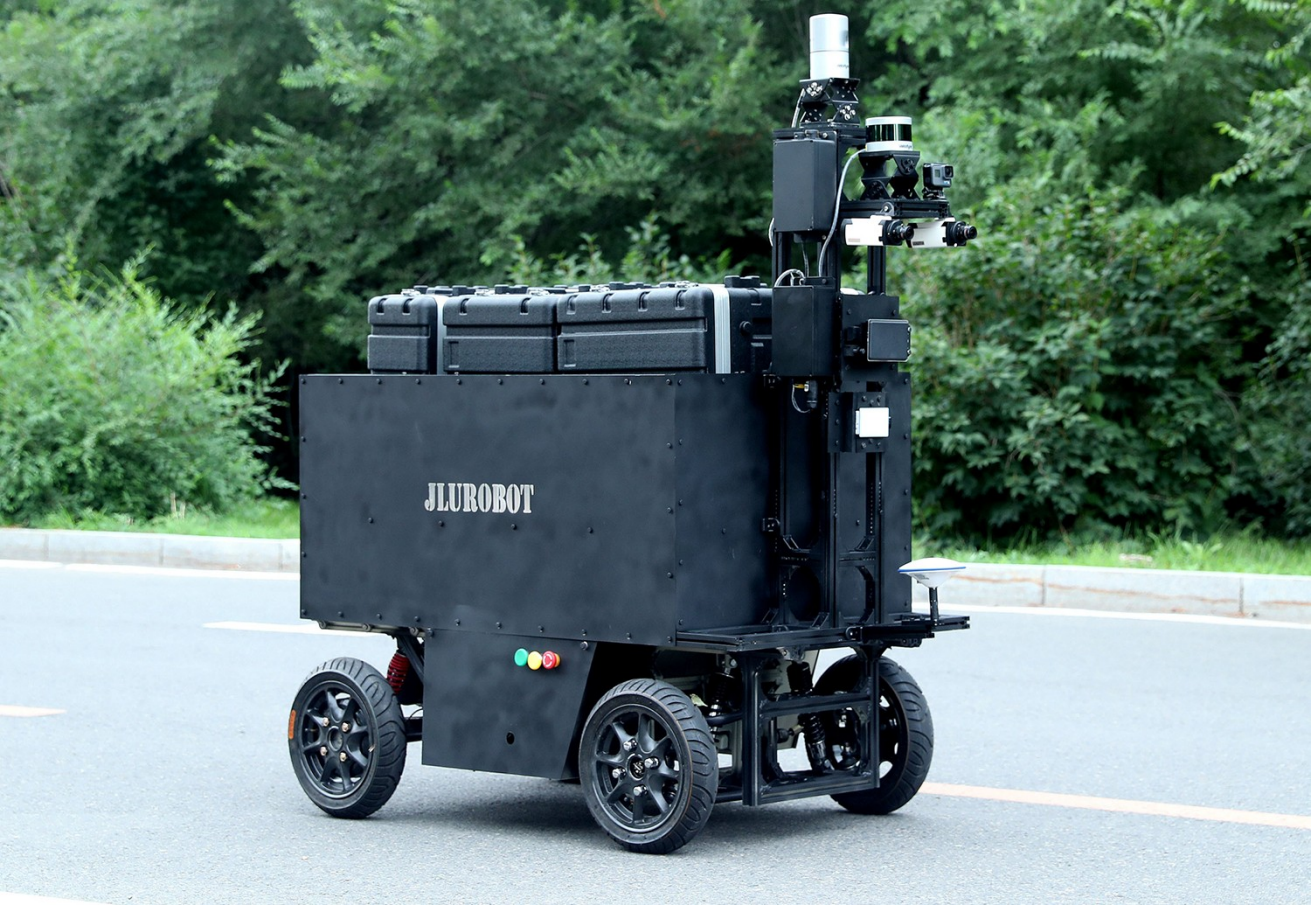
The JLU UGV.

Over the past decade, UGVs has gone from being laboratory curiosities to functional machines that are closely related to human life. UGVs can be applied to mining areas, parks and warehouses for different purposes. Specifically, object detection is a key technique of UGVs. Our research team has created a Jilin University (JLU) UGV with environmental perception, precise positioning and path planning functions. Our JLU UGV is illustrated in Fig 1.

Recently, deep learning technology has made great progress in object detection [1–4]. In particular, excellent results are obtained in the detection of pedestrians, bikes, cars and buses. Hand-engineered feature-based methods [5–7] and deep learning network-based methods [8–11] are two kinds of mainstream object detection methods.

The scale-invariant feature transform (SIFT) [12], histogram of oriented gradients (HOG) [13] and deformable part models (DPMs) [14] are famous hand-engineered feature-based methods. Invariant features can be sampled from the images using the SIFT method. Furthermore, different views of an object or scene are matched by the SIFT features. The appearance and shape of local objects in images can be extracted through the HOG method. In general, the HOG or SIFT features are applied to sample the features of objects in the DPM method.

Recently, deep learning network-based methods have obtained excellent detection results. Specifically, OverFeat [15] and multicolumn deep neural networks [16] have obtained better classification results than traditional object detection methods. Experience is applied to develop the training model of traditional methods such as the DPM and HOG method; thus, the adaptability of traditional methods is poor. Nevertheless, deep learning network-based methods can be suitable for various objects based on the same model.

Excellent classification accuracy and localization have been achieved by deep learning methods with high-quality region proposals [17, 18]. Region proposal-based object detection methods are illustrated as follows. Good object detection results are obtained by the region-based convolutional neural network (R-CNN) [19]. Additionally, the detection performance of the R-CNN is improved in the Fast R-CNN [20]. Furthermore, the quality of region proposals is enhanced in Faster R-CNN [21]. The selective search (SS) [22] is replaced by a region proposal network (RPN) to accelerate the generation speed of region proposals. In Faster R-CNN, an RPN is applied to generate region proposals. The RPN is a type of fully convolutional network (FCN) [23].

From the description of the aforementioned object detection methods, we can see that three drawbacks of Faster R-CNN need to be addressed. First, the lower and higher information is not integrated with the output features. Additionally, the scale for the RPN pooling method is fixed. In other words, the pooling method is not suitable for objects of different sizes. Therefore, the feature sampling ability of the RPN is affected. Second, the classification loss function is not sufficiently trained on the negative samples. Moreover, the coefficients for classification and the regression loss function are irrational. Thereafter, the training performance of the RPN is not satisfactory. Third, the softmax method is not adapted to classify the positive and negative proposals. As a result, the classification ability is not good. Therefore, the performance of the RPN needs to be improved.

Three improvements are developed in this paper. First, the synthesized feature sampling strategy is introduced to integrate the different layer features with the output feature. In addition, multiple pooling sizes are added to the RPN. Therefore, the RPN can adapt to objects with different shapes, and the feature sampling ability of the RPN is enhanced. Second, the expression of classification is improved to train the negative samples. Specifically, additional parameters are added to the classification and regression loss function. Furthermore, the grey wolf optimizer (GWO) is used to optimize the parameters of the improved loss function in the RPN. As a result, the performance of the loss function is improved. Third, the softmax function is replaced by a support vector machine (SVM) to improve the classifier. Additionally, the parameters of SVM are optimized by the genetic algorithm (GA). Therefore, the classification performance of the RPN is improved. As a result, the object detection ability of Faster R-CNN is strengthened.

Because some parameters of the RPN are designed based on prior knowledge, the generalizability of the RPN is inadequate. In general, we can solve this problem by replacing the fixed value with variables. Therefore, the exploration space of the RPN is expanded. The boosted RPN (BRPN) is introduced based on these reasons. However, obtaining the optimal parameter values of the BRPN is a nondeterministic polynomial-time) hard (NP-hard) problem. Therefore, the GA and GWO are applied to the BRPN. From the experimental results, we can see that our methods are effective.

The following part will introduce related work. Then the original GWO will be introduced in the literature review part. After that, our approach and contributions are described in detail. Then the experimental setup and result analysis are shown seriously. At last, we conclude the advantage and the limitation of our proposed method, and prospect the future research direction.

## Related work

In this section, several well-known deep learning-based methods are presented. An R-CNN is designed to model the relationship between object detection and image classification. First, approximately 2000 region proposals are generated using the SS method. Second, a pretrained CNN [24–27] is applied to extract the convolutional features of each region proposal. Third, the output features are classified by the SVM method. The object detection mean average precision (mAP) is improved by the RPN. Nevertheless, the processing speed is the bottleneck for the RPN.

The object detection ability of the RPN is improved by Fast R-CNN. In the training stage, the locations are optimized, and the region proposals are classified. Specifically, the region of interest (ROI) pooling method is applied to sample the features. As a result, the improved pipeline of sequentially trained tasks is used to improve the Fast R-CNN training effect. Nevertheless, the region proposal generation speed is affected by using the SS method. This problem is solved by Faster R-CNN. The RPN method is developed to reduce the time of region proposal generation.

In a multiregion CNN (MR-CNN) [28], the bounding box regression scheme is used to improve the object detection ability. The semantic segmentation-aware features are extracted through a deep MR-CNN, and the bounding boxes are evaluated twice. The Inside-Outside Net (ION) [29] extracts the inside and outside the ROI information. Skip pooling [30] is applied to achieve multiple abstraction levels and scales. HyperNet [31], performs synthesized object detection and region proposal generation tasks. Then it obtains good object detection results on the PASCAL VOC 2007 and 2012 datasets. The locations of objects in the image and their orientations on the ground plane are generated simultaneously by the FRCNN+Or [32] method. In the field of autonomous driving, Mono3D [33] is applied to perform 3D object detection from a single monocular image. The proposal generation step is the main improvement to Mono3D. A base feature extraction region is defined in the regionlet [34]. Mono3D organizes the regionlets into small groups with stable relative positions. Consistently, better joint object localization and viewpoint estimation are provided in DPM-VOC+VP [35] to improve the 3D object detection ability. Multilabel classification with partial labels [36] introduces a new classification loss that exploits the proportion of known labels per example. This approach allows the use of the same training settings as when learning with all the annotations. The stereo R-CNN [37] extends Faster R-CNN for stereo inputs to simultaneously detect and associate objects in left and right images. The sparse and dense semantic and geometric information in stereo imagery is fully exploited by this method. The generalized intersection over union (GIoU) [38] is designed to address the weaknesses of the IoU by introducing a generalized version as both a new loss and a new metric.

Recently, meta-heuristics algorithms, such as monarch butterfly optimization (MBO) [39], Slime mould algorithm (SMA) [40], Moth search algorithm (MSA) [41] and Harris Hawks Optimizer (HHO) [42], are popular to solve optimization problems. MBO is inspired by simplifying and idealizing the migration of monarch butterflies. The positions of the monarch butterflies are updated by offsprings and butterfly adjusting operator. SMA applies adaptive weights to simulate the process of producing positive and negative feedback of the propagation wave of slime mould based on its oscillation mode in nature. MSA uses the phototaxis and Levy flights of the moths to build up the general-purpose optimization method. HHO is inspired by the surprise pounce of Harris hawks. The algorithm mathematically mimics the dynamic patterns and behaviors witch based on the dynamic nature of scenarios and escaping patterns of the prey.

In the PBLS_SRPN [43], a particle swarm optimization (PSO) and bacterial foraging optimization (BFO)-based learning strategy (PBLS) is applied to improve Faster R-CNN. However, multiple pooling sizes are not considered. Additionally, the expression of the classification and regression loss function is not optimized jointly. A detailed comparison is shown in our improvements of this paper. Significant experimental results are achieved by our proposed method.

## Literature review

### Grey wolf optimizer

**Fig 2:**
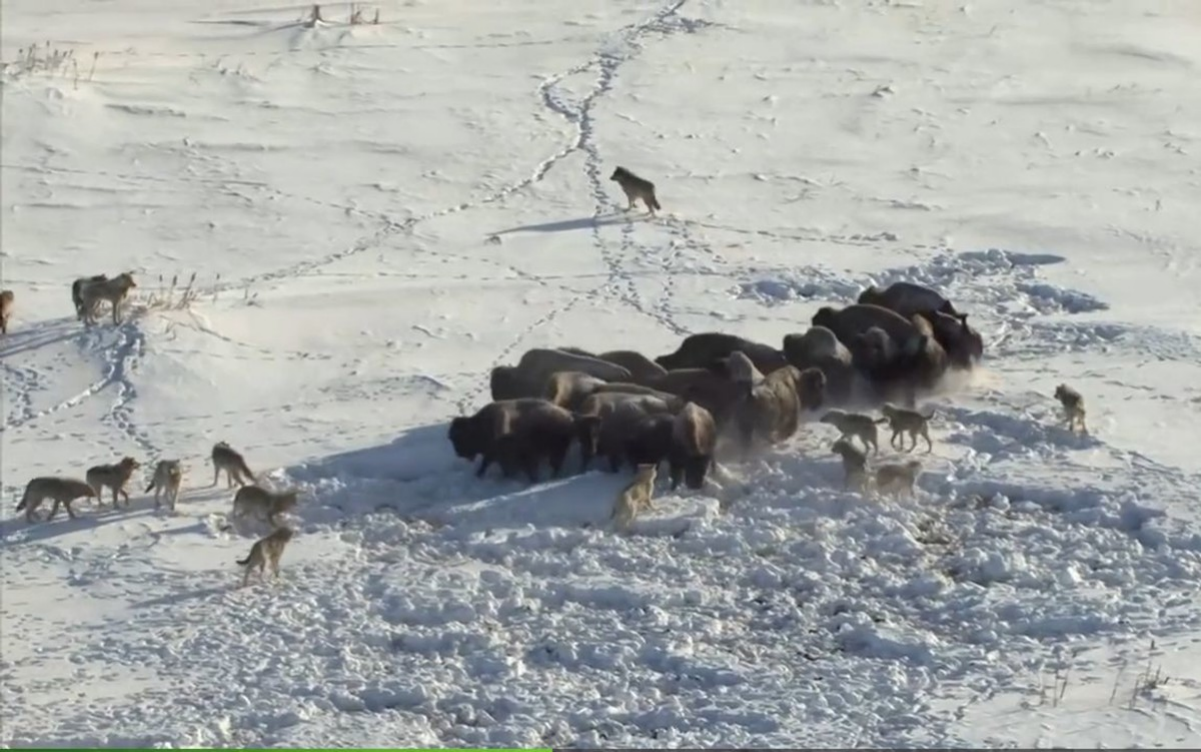
The hunting process of grey wolves.

The GWO [44] is a metaheuristic optimization method [45]. The leadership hierarchy and hunting mechanism of grey wolves is mimicked by the GWO algorithm. The hunting process for grey wolves is shown in Fig 2.

Specifically, excellent results are obtained by this method in solving optimization problems. Each grey wolf is an optimization solution in the problem field. Four types of grey wolves, alpha (*α*), beta (*β*), delta (*δ*), and omega (*ω*) wolves, are employed to simulate the leadership hierarchy. *α* represents the most suitable solution. *β* and *δ* represent the second and third best solutions, respectively. The rest of the candidate solutions are presented as *ω*. Encircling prey, attacking prey and searching for prey are the three main hunting steps of the GWO. The mathematical model for the encircling behavior is proposed in the following expressions:

$$\begin{array}{l} \vec{D}=\left|\vec{C}\cdot {\vec{X}}_{p(t)}-{\vec{X}}_{\left(t\right)}\right|\\ \vec{X}(t+1)={\vec{X}}_{p(t)}-\vec{A}\cdot \vec{D} \end{array}$$ (1)

where t represents the current iteration; the variables $\vec{A}$ and $\vec{C}$ are coefficient vectors; variable ${\vec{X}}_{p}$ is the position vector of the prey; and $\vec{X}$ is the position vector of a grey wolf. The equations for $\vec{A}$ and $\vec{C}$ are expressed as follows:

$$\begin{array}{l} \vec{A}=2\vec{\alpha }\cdot {\vec{r}}_{1}-\vec{\alpha }\\ \vec{C}=2\cdot {\vec{r}}_{2} \end{array}$$ (2)

where the value of $\vec{\alpha }$ is linearly decreased from 2 to 0 as the algorithm is iterated and the vectors *r*_1_ and *r*_2_ are in [0, 1].

The expression for encircling prey is shown as follows:

$$\begin{array}{l} {\vec{D}}_{\alpha }=\left|{\vec{C}}_{1}\cdot {\vec{X}}_{\alpha }-\vec{X}\right|,{\vec{D}}_{\beta }=\left|{\vec{C}}_{2}\cdot {\vec{X}}_{\beta }-\vec{X}\right|\\ {\vec{D}}_{\delta }=\left|{\vec{C}}_{3}\cdot {\vec{X}}_{\delta }-\vec{X}\right|\\ {\vec{X}}_{1}={\vec{X}}_{\alpha }-{\vec{A}}_{1}\cdot ({\vec{D}}_{\alpha }),{\vec{X}}_{2}={\vec{X}}_{\beta }-{\vec{A}}_{2}\cdot ({\vec{D}}_{\beta })\\ {\vec{X}}_{3}={\vec{X}}_{\delta }-{\vec{A}}_{3}\cdot ({\vec{D}}_{\delta })\\ \vec{X}(t+1)=\frac{{\vec{X}}_{1}+{\vec{X}}_{2}+\vec{X_{3}}}{3} \end{array}$$ (3)

The GWO has some variants, such as GWOCMALOL [46], IGWO [47], EGWO wrapped ELM [48], GWO-KELM [49]. The covariance matrix adaptation evolution strategy (CMAES), levy flight mechanism, and orthogonal learning (OL) are applied into GWOCMALOL to solve the structural defects and uncertain performance problem. Random local search around the optimal grey wolf in beta grey wolves, and random global search in omega grey wolves are introduced in IGWO to improve the stochastic behavior, and exploration capability of grey wolves. EGWO wrapped ELM combines the EGWO with the extreme learning machine (ELM) to identify the PQ poisoned patients. GWO-KELM adopts GWO to construct an effective kernel extreme learning machine (KELM) model for bankruptcy prediction. In this paper, the classic GWO is applied to our proposed methods. In the future, we will consider to apply other variants to improve the optimization ability.

## Our approach

### Overview

**Fig 3:**
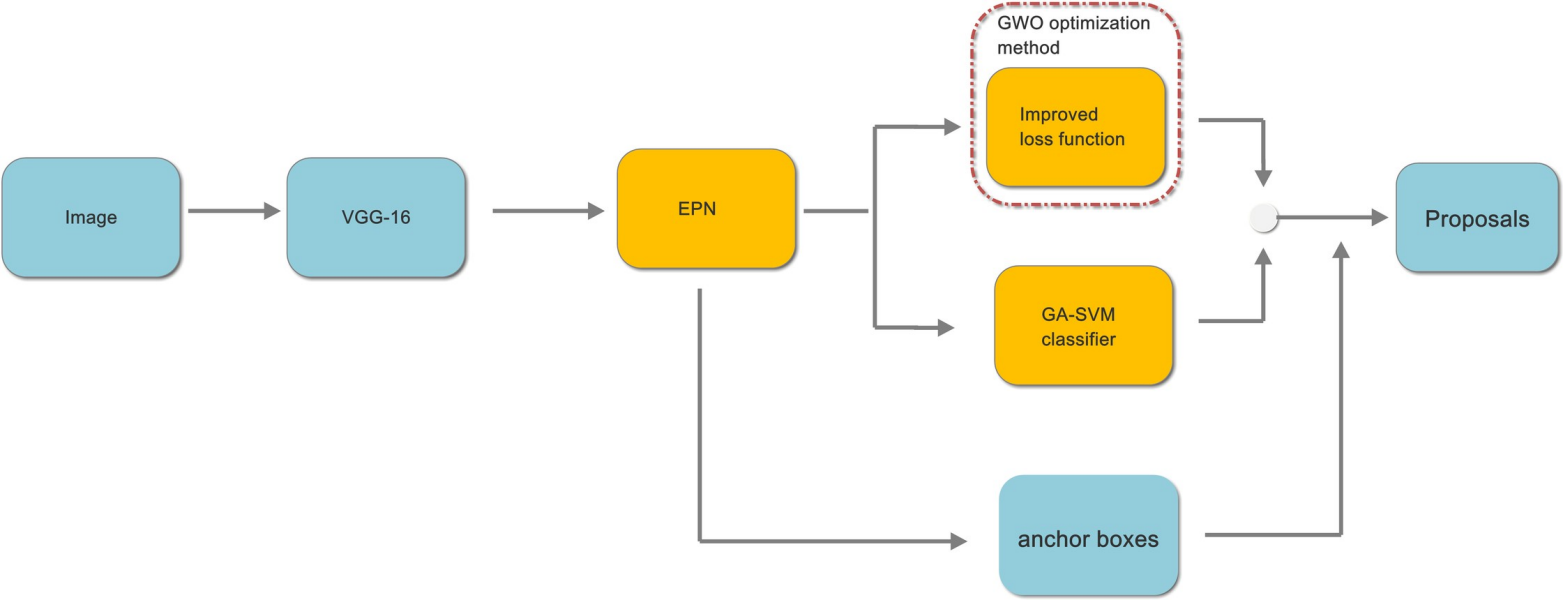
The general structure of BRPN.

The pretrained 16-layer Visual Geometry Group (VGG-16) model is applied to sample the features of the input image. In addition, Conv1, Conv2, Conv3, Conv4 and Conv5 represent the convolutional layers of VGG-16 [50]. The general structure of BRPN is illustrated in Fig 3.

From Fig 3, we can see there are three improvements in our proposed method. First, a novel enhanced pooling network is designed to sample the features of the input image in this paper. Second, the softmax classifier is replaced by an SVM classifier. In addition, the GA is used to optimize the parameters of the SVM. Third, the expression of the classification and regression loss function is improved. In addition, the parameters of the loss function are optimized by the GWO.

### A novel enhanced pooling network

Because only the last convolutional features are included in the output features for RPN, the lower feature information is not combined with the output information. Therefore, the small object detection ability of Faster R-CNN is not satisfactory. The synthesized feature sampling strategy is introduced in the enhanced pooling network (EPN).

**Fig 4:**
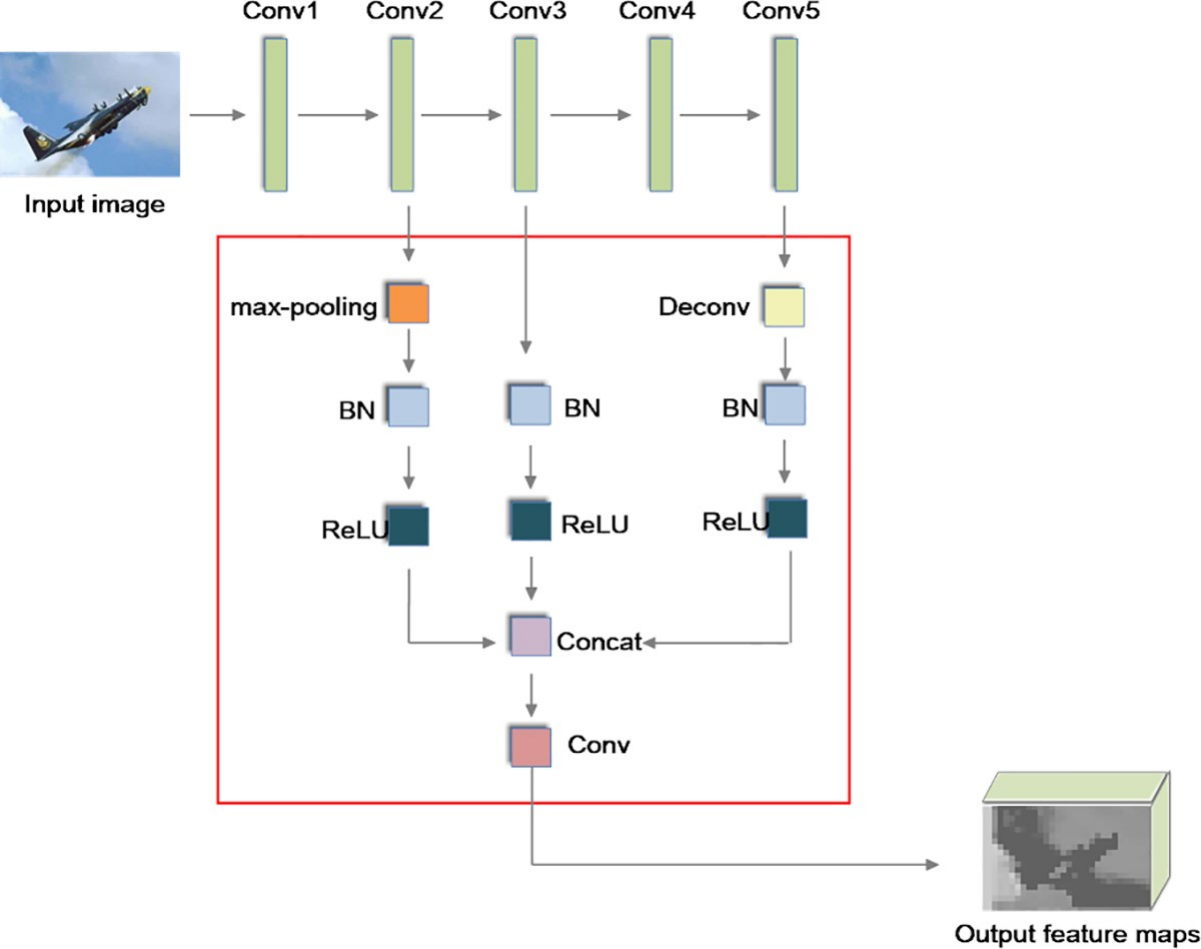
The network of the synthesized feature sampling strategy.

From Fig 4, we can see that the output feature is combined with the different layers in our synthesized feature sampling strategy. Thereafter, the output feature includes the information of the lower and higher layers. Compared with PBLS_SRPN, the sampling ability of the low convolutional layer is enhanced by this improvement. In other words, the small object detection performance of Faster R-CNN is improved.

At the beginning of Faster R-CNN, the short side of the input image is changed to 600 pixels. However, the aspect ratio of the input image is not modified. As a result, the shape of the feature map is different. Therefore, the shape of the output feature map of VGG-16 is not fixed. Specifically, the width and length of feature maps vary greatly. Nevertheless, the scale for the RPN pooling method is fixed at 7×7. Therefore, the pooling method of the RPN is not suitable for different sizes of input objects.

**Fig 5:**
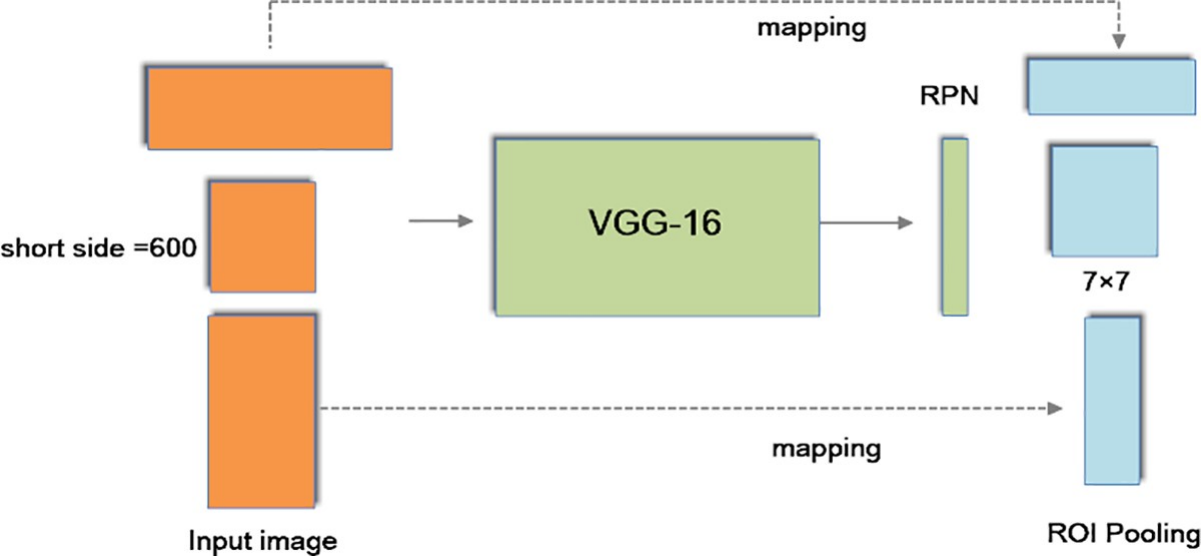
The enhanced pooling strategy.

As shown in Fig 5, we can add more ROI pooling sizes to the RPN; thus, the different shapes of the input objects can be processed very well. According to this concept, our EPN is developed for this work.

**Fig 6:**
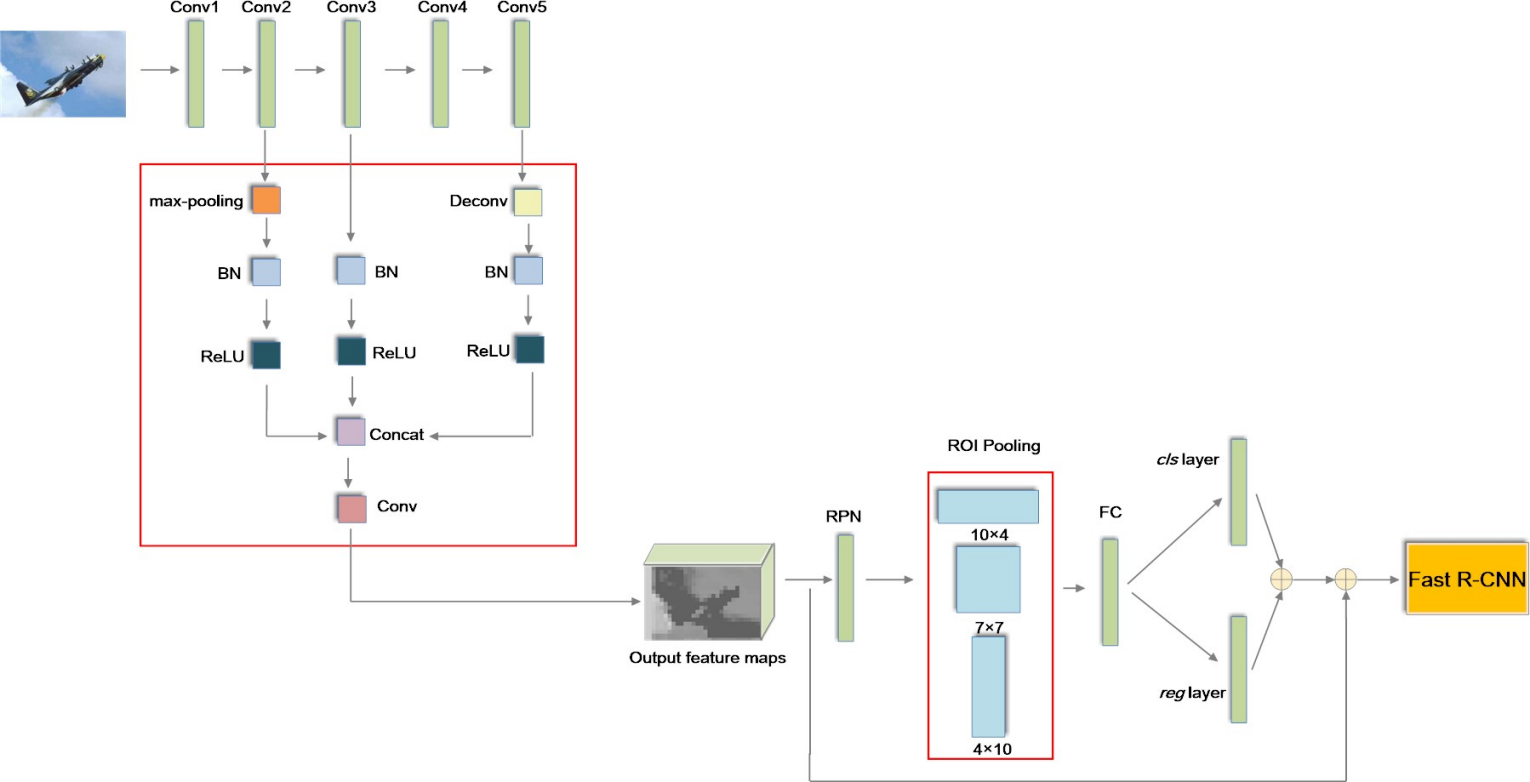
The framework of the EPN.

From Fig 6, we can see that two pooling methods of sizes 10×4 and 4×10 are applied in our EPN. The objects with heights far smaller than their widths are detected by 10×4 pooling. In addition, objects with widths far smaller than their heights are detected by 4×10 pooling. In this way, an irregular image can be detected effectively. Theoretically, we can use more pooling sizes to adapt to more shapes of input objects. However, it will cost more computation time. For a real object detection system, this is not acceptable. Moreover, different dimensions of pooling methods can be fused by using a flattened layer to make the pooled feature map into one-dimensional vectors. Considering the processing speed, the number of parameters is reduced by dimension reduction for each pooled feature map, and the vectors are sent to the fully connected layer by the concatenation layer.

Through the experimental results, we can see that the detection performance of our proposed method is improved. Thus, our improvements are effective.

### A novel GA-SVM classifier

In this paper, the SVM classifier with a radial basis function (RBF) kernel is applied to distinguish the foreground region boxes and the background region boxes. The RBF kernel of the SVM contains parameters *C* and *γ*. Nevertheless, there are no parameters for the softmax function. Therefore, the SVM algorithm has more optimization potential than the softmax method. In this paper, the GA-SVM with strong fitting ability is designed to replace the softmax method. From the experimental results, we can see that the performance of the SVM with fixed parameters is worse than that of the softmax method. However, the classification results of the GA-SVM are better than those of the softmax method. In other words, our improvements are effective.

**Fig 7:**
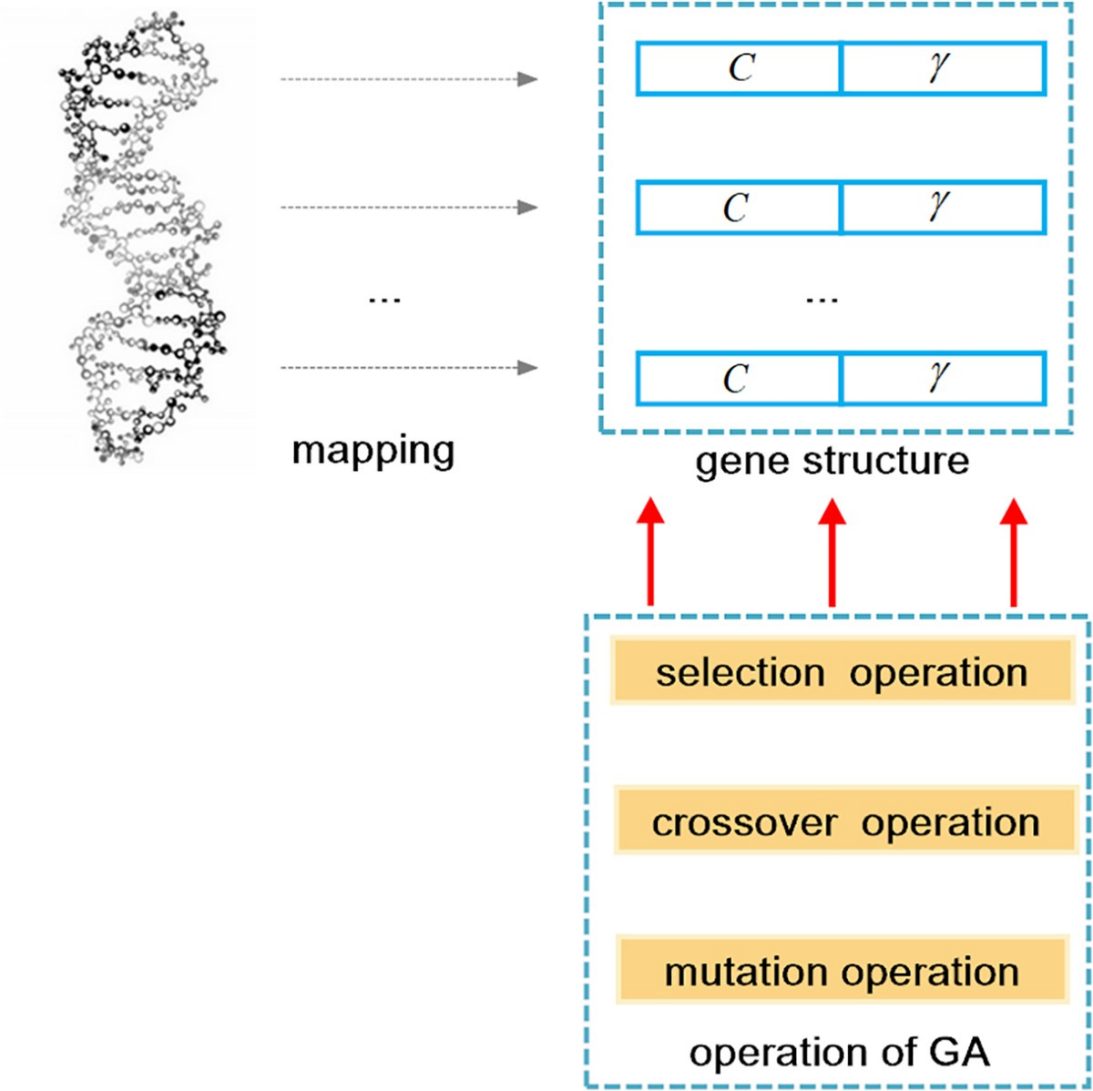
The relationship between the GA and the parameters.

To improve the performance of the SVM, we need to train *C* and *γ* of the RBF kernel. In this work, these two parameters are optimized by the GA. The relationship between the GA and the parameters are shown in Fig 7.

**Fig 8:**
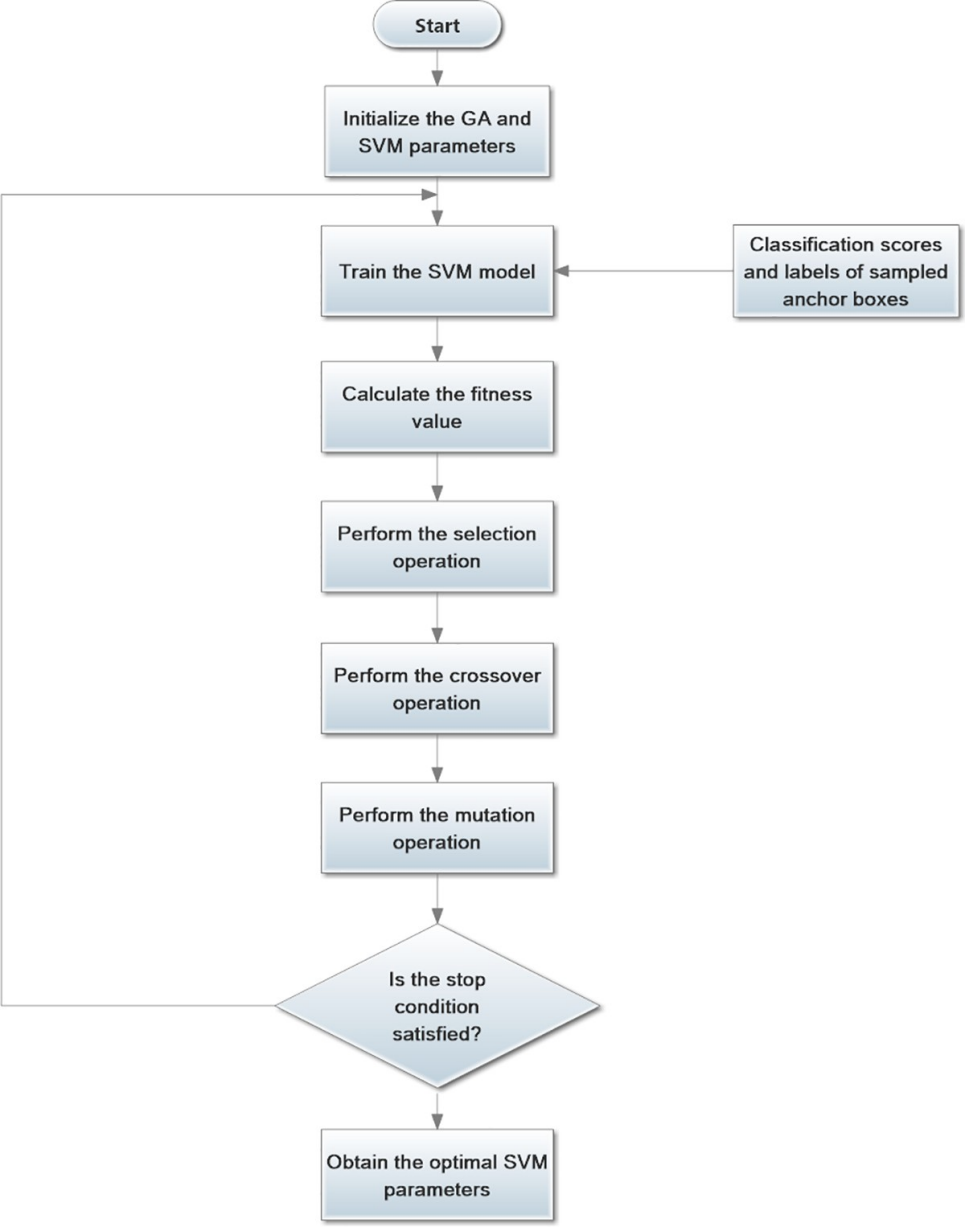
The process of SVM parameter optimization based on the GA.

The parameters of the SVM are optimized by the GA. Therefore, parameters *C* and *γ* are represented as a gene. In other words, our target is to find the best gene. The parameters *C* and *γ* are generated by the two dimensions of the gene. The fitness function is used to evaluate the performance of each gene. The values of *C* and *γ* are updated through the calculated results of the fitness function. The parameters of the SVM optimization process by the GA are shown in Fig 8.

The GA and SVM parameter are randomly initialized at the beginning of the optimization process. The SVM is trained by the anchor boxes that are generated by the BRPN. In addition, the number of positive samples must equals the number of negative samples. Because, in general, there are fewer positive samples than negative samples, some negative samples are used to complement the positive samples. The gene is updated according to the fitness calculation results. If the maximum number of iterations of the GA is reached or the fitness value satisfies the threshold, then the optimal values of parameters *C* and *γ* have been obtained; [0.01, 35,000] and [0.0001, 32] are the ranges of parameters *C* and *γ*, respectively. In the PBLS_SRPN, the parameters of the SVM are optimized by BFO. Because the computational complexity of the BFO algorithm is high, the training time is affected. In this paper, the GA is applied to optimize the SVM.

Because the values of *C* and *γ* are randomly initialized at the start of the optimization process, the number of iterations of the GA is too high. In other words, the values of parameters *C* and *γ* converge after many optimization iterations. The operation time of the BRPN should be slowly decreased. The iteration expression of the GA is described as follows:

$$n_{\mathrm{iteration}\_\mathrm{GA}}=N_{\mathrm{iteration}\_\mathrm{GA}\_\mathrm{total}}\times \frac{M_{\mathrm{iteration}\_\mathrm{BRPN}\_\mathrm{total}}-m_{\mathrm{iteration}\_\mathrm{BRPN}}}{M_{\mathrm{iteration}\_\mathrm{BRPN}\_\mathrm{total}}}$$ (4)

where *n*_iteration_GA_ is the GA operation time for one iteration of the BRPN; *N*_iteration_GA_total_ is the total operation time of the GA; *m*_iteration_BRPN_ is the current operation time of the BRPN; and *M*_iteration_BRPN_total_ is the total operation time of the BRPN.

From Eq (4), we can see that *m*_iteration_BRPN_ = 1 at the start of the BRPN. Then, the value of $\frac{M_{\mathrm{iteration}\_\mathrm{BRPN}\_\mathrm{total}}-m_{\mathrm{iteration}\_\mathrm{BRPN}}}{M_{\mathrm{iteration}\_\mathrm{BRPN}\_\mathrm{total}}}$ is close to 1. The variable *n*_iteration_GA_ approximately equals *N*_iteration_GA_total_. The value of *m*_iteration_BRPN_ is increased while the value of *n*_iteration_GA_ decreases. Thereafter, the GA iteration times are small at the end of the training phase. In other words, the training time of the GA-SVM is effectively reduced. Therefore, Eq (4) satisfies our requirement.

### Improved classification and regression loss function

The classification loss function $L_{cls}\left(p_{i},p_{i}^{*}\right)$ of Faster R-CNN is not designed rationally. Detailed information is presented in the following paragraphs. The equation for the classification loss *L*_*cls*_ is as follows:

$$L_{cls}(p_{i},p_{i}^{*})=-p_{i}^{*}\log(p_{i})-(1-p_{i}^{*})\log(1-p_{i})$$ (5)

The ground-truth label $p_{i}^{*}$ equals 1 if the anchor box is positive and 0 if the anchor box is negative. If the ground-truth label $p_{i}^{*}$ equals 1, then the *i*th anchor box is classified correctly. Therefore, the value of −log(*p*_*i*_) is small. Moreover, if the ground-truth label $p_{i}^{*}$ equals 0, the value of −log(1−*p*_*i*_) is large. Sometimes the value of −log(1−*p*_*i*_) is not much larger than that of −log(*p*_*i*_). Nevertheless, the number of positive samples is far less than that of negative samples in general. Therefore, the training effect of the negative samples should be more than that of the positive samples. In this paper, the classification loss function is improved to reduce the effect of the positive samples. The improved classification loss function is developed as follows:

$$L_{cls}(p_{i},p_{i}^{*})=-e^{-\eta (-p_{i})}p_{i}^{*}\log(p_{i})-e^{-\eta p_{i}}(1-p_{i}^{*})\log(1-p_{i})$$ (6)

where the coefficient *η* is applied to adjust the function.

**Fig 9:**
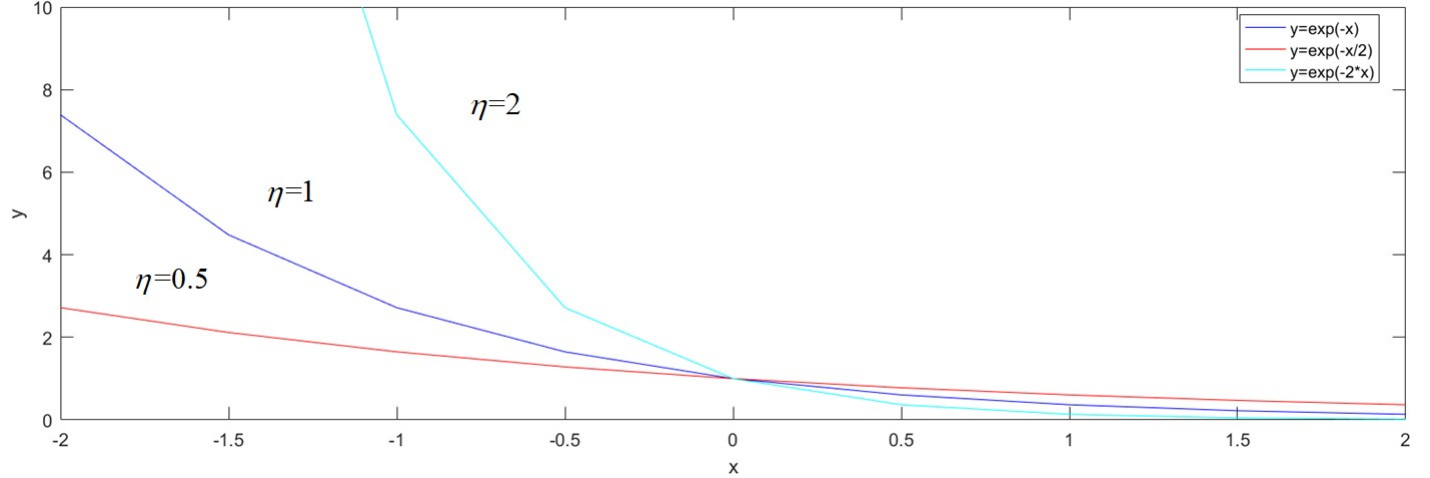
The graph of $e^{‐\eta p_{i}}$ with different values of *η*.

If $p_{i}^{*}=0$, then the value of $e^{-\eta (-p_{i})}$ is greater than 1. Therefore, the training effect of the negative samples is increased. Moreover, if $p_{i}^{*}=1$, then the value of $e^{‐\eta p_{i}}$ is lower than 1. Thus, the training effect of positive samples is seriously reduced. As a result, the negative samples are learned more than the positive samples. In other words, the training performance of the RPN is enhanced. From Fig 9, we can see that the curve of $e^{‐\eta p_{i}}$ is affected by different values of *η*. Therefore, the value of the coefficient *η* is important to the training performance. However, the coefficient *η* is hard to design. In this paper, this coefficient is optimized by the swarming intelligence function. The definition of the regression loss function is as follows:

$${\mathrm{smooth}}_{L_{1}}(x)=\left\{ \begin{array}{l} 0.5x^{2}\;if\;\left|x\right|<1\\ \left|x\right|-0.5\;\mathrm{otherwise}, \end{array}\right.$$ (7)

The smooth *L*_1_ loss function is constructed based on the *L*_1_ and *L*_2_ loss functions. The *L*_1_ and *L*_2_ loss functions are defined as follows:

$$\begin{array}{l} L_{1}(x)=\lambda \left|x\right|\\ L_{2}(x)=\theta x^{2} \end{array}$$ (8)

where *λ* and *θ* are the influencing parameters. From Eq (8), we can see that the descending speed of the *L*_2_ loss function is slower than that of the *L*_1_ loss function. As a result, the convergence effect of the *L*_1_ loss is better than that of the *L*_2_ loss. The smooth *L*_1_ loss function is represented by the *L*_2_ loss function between -1 and 1, while the smooth *L*_1_ loss function is the *L*_1_ loss function in other areas. Thereafter, the features of the *L*_1_ and *L*_2_ loss functions are included in the smooth *L*_1_ loss function. The smooth *L*_1_ loss function is a special case of the Huber loss function. Specifically, the *L*_2_ loss function plays an important role if the value of *x* is in the range of [−*λ*, *λ*]. However, the *L*_1_ loss function occupies a leading position in other ranges. Therefore, the calculation effectiveness of the Huber loss function depends on the value of *λ*. Additionally, 0.5 may not be the optimal value; 0.5 is represented by the variable *θ*. The improved loss function is described as follows:

$$H(x)=\left\{ \begin{array}{l} \theta x^{2}\;if\;\left|x\right|<\lambda \\ \lambda (2\theta \left|x\right|-\theta \lambda )\;\mathrm{otherwise}, \end{array}\right.$$ (9)

where *λ* and *θ* are the influencing factors. The parameters *λ* and *θ* can be assigned by the swarming intelligence function.

Consequently, the performance of the classification loss function is enhanced by strengthening the negative sample training effect. Additionally, the coefficients of the classification and regression loss function are suitable to be trained by the swarming intelligence function.

### Loss function parameters optimization with the GWO

The factors *λ* and *θ* are set to 1 and 0.5 in the regression loss function based on experience. Nevertheless, these values are not globally optimal. In addition, the improved classification loss function contains the factor *η*. Finding the best values for influence factors *λ*, *θ* and *η* is not easy. The grid search method is the conventional way to find the best values of *λ*, *θ* and *η*. However, the value of the search step is difficult to define in the grid search method. If the value of the search step is not suitable, then the operation results are not optimal. In other words, the exploitation ability is affected by the search step.

**Fig 10:**
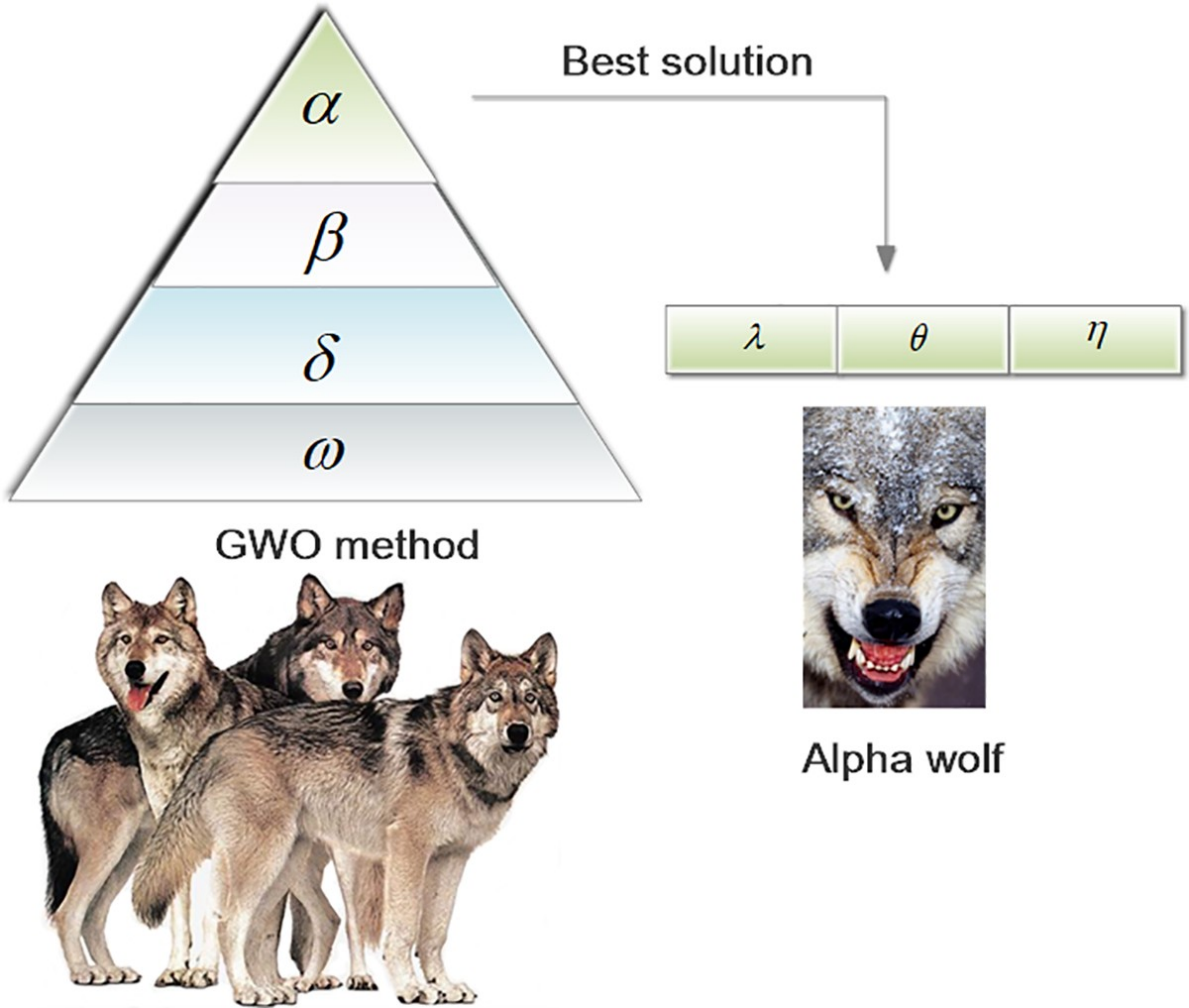
The relationship between the GWO and loss function coefficients.

In this paper, the GWO is introduced to optimize the influencing factors. The loss function coefficients are determined by two types of parameters. Each grey wolf is defined by three parameters, *λ*, *θ* and *η*. Therefore, the changes in parameters *λ*, *θ* and *η* are represented by grey wolves. In addition, the ability of each grey wolf is calculated by the fitness function. In this work, our improved loss function is the fitness function. The leaders are called *α* and include males and females. The *α* wolves are mostly responsible for making decisions about hunting, finding a place for sleeping, determining the time to wake, and so on. The second level in the hierarchy of grey wolves is *β*. The *β* wolves are subordinate wolves that help the *α* wolf with decision-making and other pack activities. The lowest ranking grey wolves are *ω* wolves. The *ω* wolves play the role of scapegoats. The last type of wolves is *δ* wolves, which are subordinate to *α* and *β* wolves but dominate *ω* wolves. Because the best solution is *α*, to find the optimal *λ*, *θ* and *η* is to find *α*. The fitness function is a way to measure the wolves. The relationship between the GWO and the loss function coefficients is shown in Fig 10.

**Fig 11:**
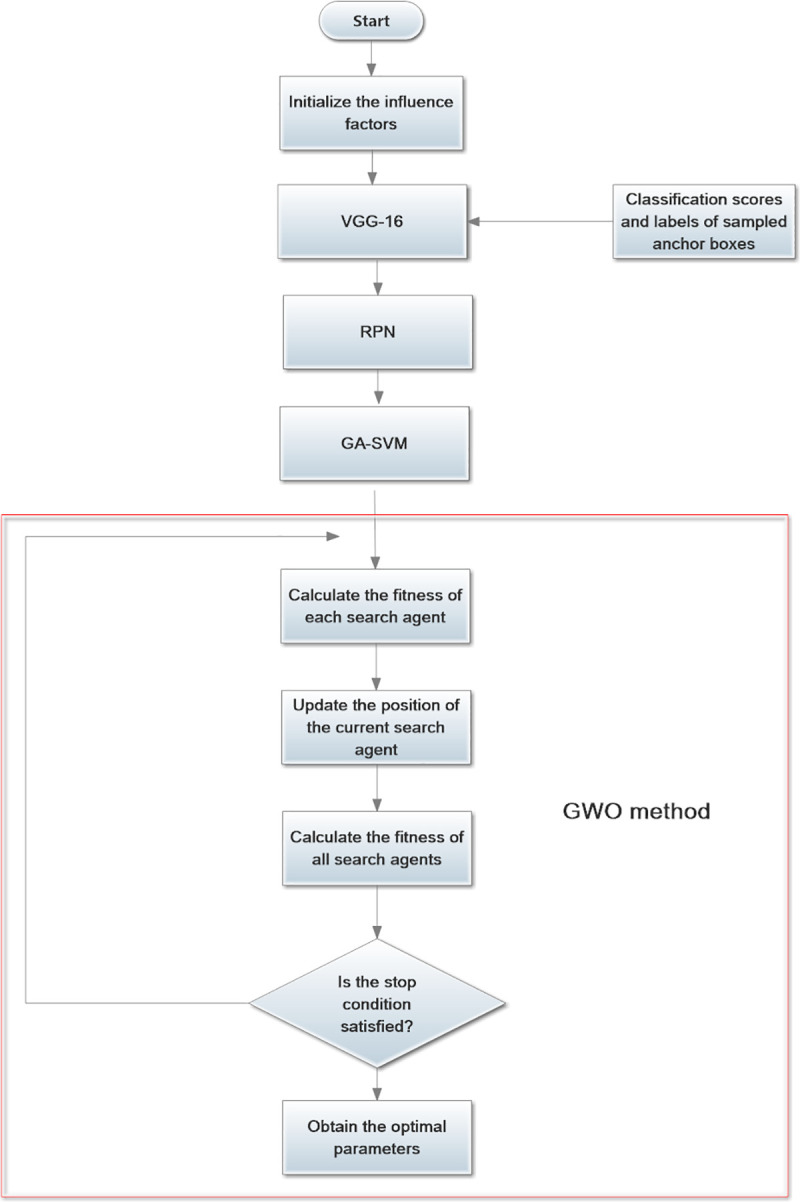
Flowchart of the parameter optimization process with GWO.

First, the parameters *λ*, *θ* and *η* are initialized. The default values of *λ*, *θ* and *η* in the loss function are 1, 0.5 and 1 at the beginning. The optimal grey wolves *α* are calculated and updated by the GWO. The best values are processed based on the fitness function. The optimization flow is finished when the operation stop condition of the GWO is satisfied. The flowchart in Fig 11 represents the BRPN parameter optimization process through the GWO.

In the PBLS_SRPN, the GA optimization process is contained in the PSO process. Thereafter, the training procedure is complicated. Nevertheless, the SVM and loss function optimization procedures are serially executed by the GA and GWO. In other words, the training process is simplified. As a result, the training time is decreased.

## Experiments

### Datasets

The PASCAL VOC 2007, 2012 [51] and KITTI [52] datasets are applied to train and test our proposed method. The comparison between our proposed method and the state-of-the art object detection methods is shown in this part. The information on our experimental datasets is presented in Table 1. Moreover, the performance of the BRPN on object detection is verified.

**Table 1:** Dataset information.

No.	Dataset	No. of categories	No. of annotated objects
1	PASCAL VOC 2007	20	24,640
2	PASCAL VOC 2012	20	27,450
3	KITTI	3	80,256

The Caffe [53] framework is used to realize our proposed method. The convolutional parameters are initialized through the pretrained VGG-16 model. The VGG-16 model contains 13 convolutional layers and 3 fully connected layers. The performance of our proposed method is evaluated based on the mAP and recall. To ensure the stability of the experimental results, we repeat each method 3 times and average the results.

### Parameter setting

The GA, GWO and BRPN parameters used during our experiments are shown in the Tables 2–4.

**Table 2:** GA parameters.

Parameter	Description	Value
*N*	Number of genes	30
*N*_*IN*_	Number of iterations	100
*N*_*C*_	Crossover rate	0.8
*N*_*M*_	Mutation rate	0.05

**Table 3:** GWO parameters.

Parameter	Description	Value
*α*	Initial value of the GWO parameter	2
*r*_1_	Random factor for the GWO	[0, 1]
*r*_2_	Random factor for the GWO	[0, 1]

**Table 4:** BRPN parameters.

Parameter	Description	Value
base_lr	Initial value of the learning rate	0.001
lr_policy	Learning rate policy: drop the learning rate in steps by a factor of gamma every stepsize iterations	"step"
gamma	Factor of the dropped learning rate	0.1
stepsize	Drop the learning rate every stepsize iterations	50,000
momentum	Weight of the previous update	0.9
weight_decay	Regularization factor	0.0005
*max_iter_rpn*	Number of BRPN execution steps	70,000
*IoU_ foreground*	IoU for the BRPN foreground proposals	[0.6, 1]
*IoU_ background*	IoU for the BRPN background proposals	[0, 0.3)
*α*	Factor for the improved loss function	1
*β*	Factor for the improved loss function	0.5
*η*	Factor for the improved loss function	1
NMS_IoU	Threshold value of the IoU method	0.65

### Experiments on the PASCAL VOC 2007 dataset

In the experiments, the proposed method named BRPN-GE only contains the EPN strategy and GA-SVM classifier improvements. The final proposed method named BRPN is based on BRPN-GE. Additionally, it includes a strengthened loss function witch the parameters is optimized by GWO.

In Table 5, the mAP of the BRPN-GE model is 78.2%, which is higher than that of the Fast R-CNN, Faster R-CNN, ION and HyperNet models. The reason includes as follows. Firstly, the output feature maps of BRPN-GE contain lower feature maps and higher feature maps. And multiple pooling sizes are added to the BRPN-GE. Therefore, the BRPN-GE model can adapt to objects with different shapes. The feature sampling ability for our proposed method is boosted through EPN improvement. Secondly, the classification capability is promoted based on the GA-SVM method. From the experimental results, improvements in the BRPN-GE model are effective.

**Table 5:** Detection results on the PASCAL VOC 2007 test set.

Approach	mAP	aero	bike	bird	boat	bottle	bus	car	cat	chair	cow	table	dog	horse	motorbike	person	plant	sheep	sofa	train	tv
Fast R-CNN	70.0	77.0	78.1	69.3	59.4	38.3	81.6	78.6	86.7	42.8	78.8	68.9	84.7	82.0	76.6	69.9	31.8	70.1	74.8	80.4	70.4
Faster R-CNN	73.2	76.5	79.0	70.9	65.5	52.1	83.1	84.7	86.4	52.0	81.9	65.7	84.8	84.6	77.5	76.7	38.8	73.6	73.9	83.0	72.6
MR-CNN	78.2	**80.3**	84.1	78.5	70.8	68.5	88.0	85.9	87.8	60.3	**85.2**	73.7	87.2	86.5	85.0	76.4	48.5	76.3	75.5	85.0	81.0
ION	75.6	79.2	83.1	77.6	65.6	54.9	85.4	85.1	87.0	54.4	80.6	73.8	85.3	82.2	82.2	74.4	47.1	75.8	72.7	84.2	80.4
HyperNet	76.3	77.4	83.3	75.0	69.1	62.4	83.1	87.4	87.4	57.1	79.8	71.4	85.1	85.1	80.0	79.1	51.2	**79.1**	75.7	80.9	76.5
PBLS_SRPN	78.9	79.7	84.6	79.2	69.7	68.9	**88.3**	**87.8**	87.6	61.8	83.7	**74.9**	86.2	86.6	85.7	**79.3**	52.2	77.5	75.5	**86.1**	82.3
BRPN-GE	78.2	78.4	83.4	78.4	68.9	68.4	86.1	86.1	87.8	61.7	83.4	74.4	86.3	85.9	84.9	78.2	51.3	77.1	76.2	85.7	82
BRPN	**79.1**	79.6	**84.7**	**79.3**	**69.9**	**69.5**	87.5	86.5	**88.1**	**62.3**	84.5	74.8	**87.7**	**86.8**	**85.8**	79	**52.4**	78.8	**77.1**	85.9	**82.8**

The best AP of each object category and mAP are highlighted in bold.

Additionally, the mAP of the BRPN model (79.1%) is the best. Compared with the BRPN-GE model, this is benefit from the strengthened loss function which the parameters are optimized by GWO. Outstanding results are achieved by the BRPN on bikes, birds, boats, bottles, cats, chairs, dogs, horses, motorbikes, plants, sofas and trains. In other words, the BRPN can classify objects more accurately than the other comparison methods. The classification ability of the BRPN is improved based on our modifications.

### Experiments on the PASCAL VOC 2012 dataset

Our proposed BRPN-GE and BRPN are trained and tested on the PASCAL VOC 2012 dataset. Additionally, the VOC 2007 and VOC 2012 datasets are integrated as training data. In Table 6, a mAP of 74.3% is achieved by the BRPN-GE model. The mAPs of the Fast R-CNN, Faster R-CNN, ION, MR-CNN and HyperNet models are lower than that of the BRPN-GE model. However, the mAP of the BRPN model is higher than that of the BRPN-GE and PBLS_SRPN models. The PBLS_SRPN model achieves the highest AP on buses, cars and people. Furthermore, the Fast R-CNN model achieves the highest AP of 89.3% on cats. Obviously, the highest APs are obtained by the BRPN model on the remaining objects. In other words, our proposed BRPN model can achieve the best object detection results on the PASCAL VOC 2012 dataset. Therefore, the BRPN model is adaptive to sophisticated datasets.

**Table 6:** Detection results on PASCAL VOC 2012 test set.

Approach	mAP	aero	bike	bird	boat	bottle	bus	car	cat	chair	cow	table	dog	horse	motorbike	person	plant	sheep	sofa	train	tv
Fast R-CNN	68.4	82.3	78.4	70.8	52.3	38.7	77.8	71.6	**89.3**	44.2	73.0	55.0	87.5	80.5	80.8	72.0	35.1	68.3	65.7	80.4	64.2
Faster R-CNN	70.4	84.9	79.8	74.3	53.9	49.8	77.5	75.9	88.5	45.6	77.1	55.3	86.9	81.7	80.9	79.6	40.1	72.6	60.9	81.2	61.5
MR-CNN	73.9	85.5	82.9	76.6	57.8	62.7	79.4	77.2	86.6	55.0	79.1	62.2	87.0	83.4	84.7	78.9	45.3	73.4	65.8	80.3	74.0
ION	71.1	83.3	80.5	71.5	56.5	53	77.4	73.8	85.8	52.6	76.8	59.1	83.9	81.3	79.3	77.2	45.7	72.6	64.2	80.1	68.1
HyperNet	71.4	84.2	78.5	73.6	55.6	53.7	78.7	79.8	87.7	49.6	74.9	52.1	86.0	81.7	83.3	81.8	48.6	73.5	59.4	79.9	65.7
PBLS_SRPN	74.8	85.2	83.5	76.8	58.1	63.3	**79.5**	**80.2**	86.5	55.7	79.5	63.1	87.8	84.6	85.1	**82.1**	49.2	74.1	65.4	81.9	74.5
BRPN-GE	74.3	85.7	83.1	76.9	56.8	63.4	78.8	78	86.6	55.8	79.2	62.9	86.7	84.4	85.1	78.9	48.8	73.6	65.9	81.2	73.9
BRPN	**75.1**	**86.6**	**83.9**	**77.7**	**58.2**	**64**	79.3	77.8	87.7	**56.3**	**79.8**	**63.6**	**88**	**84.9**	**86**	80	**49.3**	**74.5**	**67.1**	**82**	**74.8**

The best AP of each object category and mAP are highlighted in bold.

### Small-object detection

As we know, small objects are composed of few pixels; therefore, small objects are difficult to detect. In the VOC 2007 and VOC 2012 dataset, birds, bottles and potted plants are small objects. Thus, our proposed methods are applied to detect these challenging objects.

**Fig 12:**
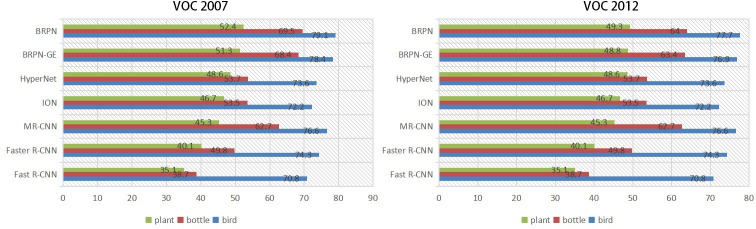
Small-object detection results on the PASCAL VOC 2007 and VOC 2012 test sets.

From Fig 12, we can see that the APs of the Fast R-CNN and Faster R-CNN models on detecting bottles, birds and potted plants are lower than that of the BRPN-GE model. The reason is that the lower feature maps and higher feature maps are synthesized with the output feature maps in the BRPN-GE model. Additionally, multiple pooling sizes are supplemented with the BRPN-GE method; therefore, the capability of small-object feature sampling of the BRPN-GE model is boosted. Furthermore, the BRPN achieves the highest AP in detecting birds, bottles and potted plants. The softmax classifier is replaced by the GA-SVM classifier with the RBF kernel, which strengthens the classification ability of the BRPN model. In addition, the GWO is applied to optimize the parameters of the classification loss functions. As a result, the classification ability of the BRPN model is further improved. In other words, the small-object detection performance of our proposed methods is enhanced.

### Analysis of the recall vs. IoU and ROC curves

**Fig 13:**
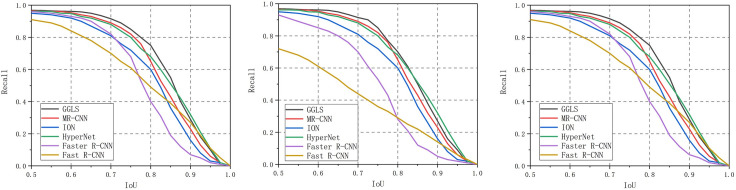
Recall versus IoU threshold on the VOC 2007 test set. Left: 150 region proposals. Middle: 450 region proposals. Right: 850 region proposals.

The current well-known object detection method is compared with our proposed method. In total, 150, 450 and 850 region proposals are applied to the BRPN. The different IoU values are applied to calculate the recall of the region proposals on the PASCAL VOC 2007 dataset. The chosen region proposals are ranked according to the scores from high to low. From Fig 13, we can see that the recall value of the MR-CNN, ION, HyperNet, Fast R-CNN and Faster R-CNN models drop quickly as the number of region proposals decreases. The quality of the region proposals in these object detection methods is poor because of their feature sampling strategy. Features from different levels are not considered in feature sampling. Therefore, the recall values are affected at the same IoU value. Multiple-layer features are synthesized based on our EPN method. Additionally, the number of pooling sizes of the BRPN is increased. Therefore, the feature sampling ability of our proposed method is enhanced. Furthermore, the foreground and background region proposals are distinguished through the GA-SVM classifier. As a result, only 150 region proposals are sufficient for our proposed method to generate valid region proposals. As the number of region proposals is reduced from 850 to 150, the recall of our proposed method is basically unchanged. The recall of our BRPN model is high, and the number of region proposals is equal to 150 when the IoU value is assigned to 0.65. Therefore, the value of the IoU threshold for the BRPN is set to 0.65. Because the object detection speed is affected by the number of region proposals, the computing speed of the BRPN can be accelerated with fewer region proposals.

**Fig 14:**
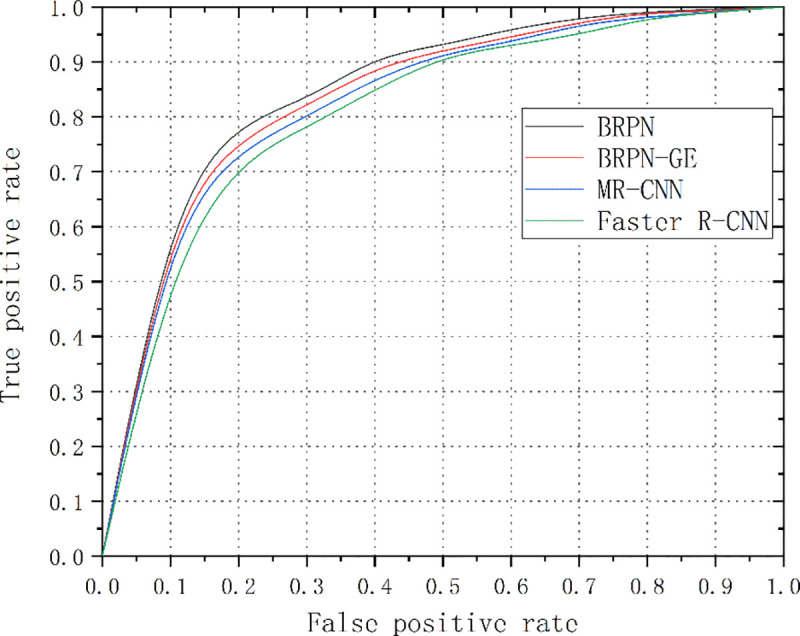
ROC curves of the compared methods.

The receiver operating characteristic (ROC) curves for the 3 best schemes and the Faster R-CNN model are presented in Fig 14. It can be seen that the BRPN, BRPN-GE and MR-CNN models consistently outperform the Faster R-CNN model. Furthermore, the BRPN-GE model achieves the best experimental results.

### Analysis of the novel EPN and training process

In our proposed EPN improvement, more ROI pooling sizes are added to the BRPN; thus, the different shapes of input objects can be processed effectively. Experiments over different pooling sizes are executed on the VOC 2007 dataset. From Table 7, we can see that a mAP of 78.6% is achieved by the BRPN model with a 7×7 pooling size. In this situation, the scale for the BRPN pooling method is fixed at 7×7. Therefore, the pooling method of the BRPN is not suitable for different sizes of input objects. Moreover, a mAP of 78.9% is achieved by the BRPN model with 7×7 and 10×4 pooling sizes. Therefore, objects with heights far smaller than their widths are detected by a pool size of 10×4. In other words, the detection capability of the BRPN is strengthened. Finally, a mAP of 79.1% is achieved by the BRPN with 7×7, 10×4 and 4×10 pooling sizes. The reason is that objects with widths far less than their heights are detected by the pool size of 4×10. As a result, the BRPN with three pooling sizes achieves the best results. In other words, the feature sampling capability of the BRPN is promoted by the novel EPN.

**Table 7:** Experimental results over the novel enhanced pooling network.

Pooling size	mAP	aero	bike	bird	boat	bottle	bus	car	cat	chair	cow	table	dog	horse	motorbike	person	plant	sheep	sofa	train	tv
7×7	78.6	79.1	84.2	78.2	69.6	69.2	87.1	85.9	87.2	62.1	83.8	74.1	86.8	86.6	85.5	78.4	52.2	78.5	76.1	85.5	82.4
7×7,10×4	78.9	79.3	84.5	78.7	69.8	69.4	87.2	86.3	87.8	62.2	84.4	74.6	87.4	86.7	85.5	78.9	52.4	78.6	76.7	85.6	82.6
7×7,10×4, 4×10	**79.1**	**79.6**	**84.6**	**79.1**	**69.9**	**69.5**	**87.5**	**86.5**	**88.1**	**62.3**	**84.5**	**74.8**	**87.7**	**86.8**	**85.7**	**79**	**52.4**	**78.8**	**77.1**	**85.9**	**82.8**

The curve of the classification loss function is affected by different values of *η*. In addition, *λ* and *θ* are the influencing factors for the improved regression function. Therefore, the values of coefficients *η*, *λ* and *θ* are optimized by the GWO. To reduce the training time, the ranges of *η*, *λ* and *θ* are restricted to (0, 2). In this section, the fluctuations of *η*, *λ* and *θ* in the VOC 2007 dataset are illustrated as follows.

**Fig 15:**
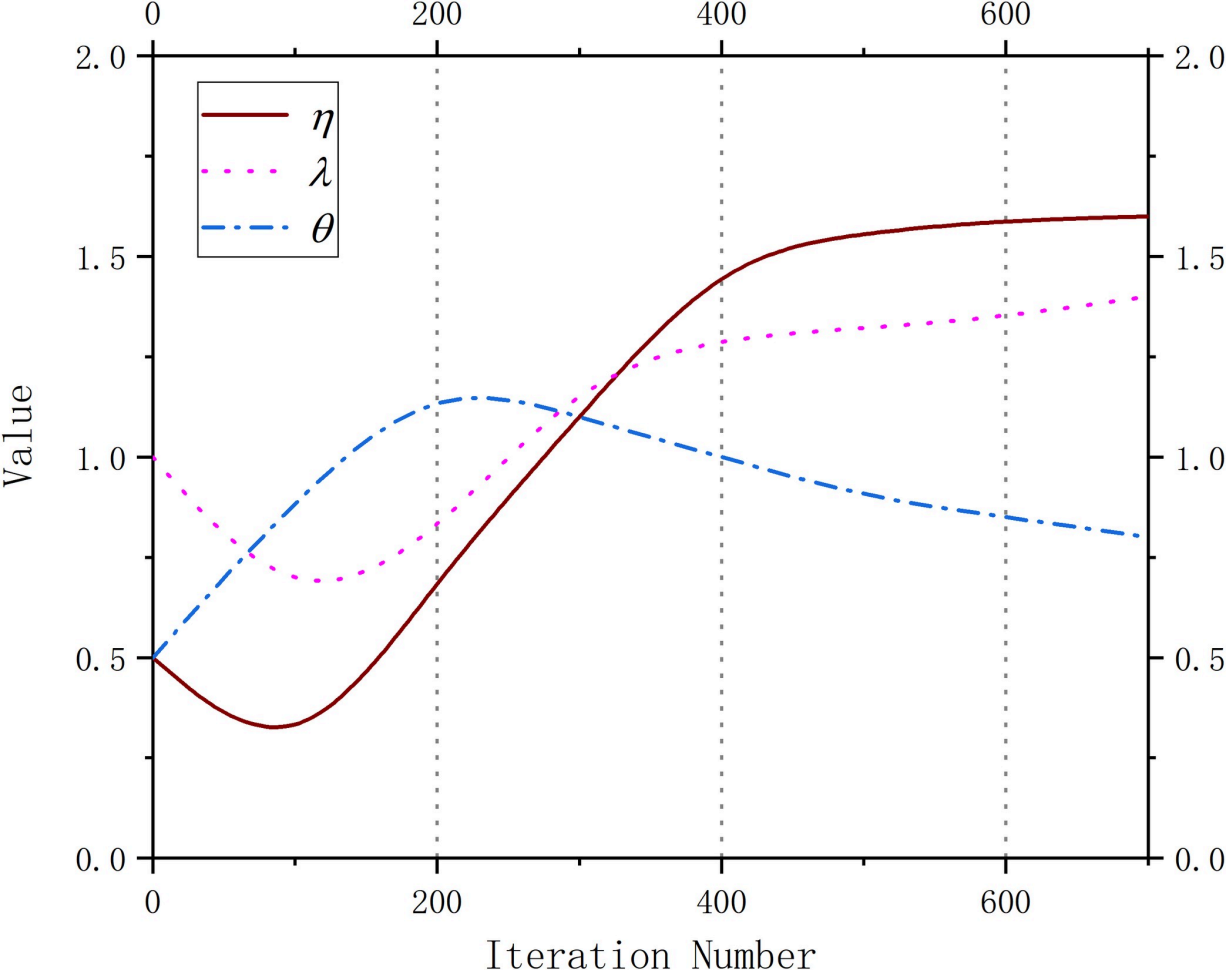
The optimization process for the different coefficients.

The initial values of *λ*, *θ* and *η* in the loss function are 1, 0.5 and 1, respectively. These coefficients are optimized by the GWO. Each grey wolf is defined by three parameters *λ*, *θ* and *η*. Therefore, the optimal solution for these parameters is to find the wolf leaders. The fitness function is a way to measure the wolves. The optimal grey wolves *α* can be calculated and updated in the GWO. From Fig 15 and Table 8, we can see that the values of *λ* and *η* are reduced when the number of iterations = 100. When the maximum number of iterations is between 100 and 200, these values are larger than before. The reason is that the GWO gradually finds better wolves from the lower-level wolves. The value of *θ* is increased before the number of iterations = 200 and then decreased when number of iterations is between 200 and 600. This fluctuation represents the process of finding the wolf leaders. Specifically, the values of *λ*, *θ* and *η* are stable in the last iteration phases, and the global optimal solution is found. Additionally, we can see that the AP continues to increase. Based on the experimental results, we can conclude that the GWO is effective in improving the classification ability of the BRPN.

**Table 8:** Runtime data for the different coefficients.

Experimental Results	*η*	*λ*	*θ*	AP
Number of iterations = 0	0.5	0.5	1	0
Number of iterations = 200	0.73	0.77	1.22	42.50%
Number of iterations = 400	1.47	1.26	1.1	67.40%
Number of iterations = 600	1.68	1.39	0.82	78.90%
Number of iterations = 800	1.69	1.41	0.79	79.10%

From Table 9, we can see that the training error of the Faster R-CNN model is 0.173 when it is trained 160,000 times. However, the training error for the BRPN is 0.08 when it is trained 800 times. Obviously, the training error of the BRPN model is better than that of the Faster R-CNN model. The reason is that the feature sampling ability of the BRPN is enhanced by introducing the synthesized feature sampling strategy. Therefore, the quality of the input data for the BRPN is strengthened. Additionally, the classification and regression loss function of the BRPN is improved. Therefore, the effect of backpropagation for the BRPN and the performance of the BRPN are improved.

**Table 9:** Experiment results of the training error.

Method	Training times	Training error
Faster R-CNN	160,000	0.173
BRPN	800	0.08

### Analysis of the novel loss function

In this part, the VOC 2007 and VOC 2012 datasets are used to show the effect of our novel loss function. The experimental results of the three situations are compared in Table 10.

**Table 10:** Experimental results over the novel loss function optimized by the GWO.

Dataset	VOC 2007			VOC 2012		
*L*_*cls*_	√	√	√	√	√	√
*L*_*reg*_(RPN)		√			√	
*L*_*reg*_(BRPN)			√			√
mAP (%)	69.4	73.2	79.1	66.2	70.4	75.1

Only the classification loss (*λ* = 0) in the loss function is presented in the first column of each group. Thus, the worst results are achieved without the bounding box regression loss function for the Faster R-CNN model. The classification loss and bounding box regression loss of the RPN are shown in the second column of each group. Because the quality of the region proposals is enhanced based on the bounding box regression loss function, the processing results of the second column for each group are better than those of the first column. In the third column of each group, the classification loss function of the BRPN is improved to reduce the effect of positive samples. As a result, the negative samples are learned more than the positive samples, and the training performance of the BRPN is enhanced. In addition, more coefficients are added to the regression loss function. Moreover, the parameters of the loss function are optimized by our GWO. Therefore, the best results are achieved by the BRPN in the third column.

### The effect of improved classification method

In this section, the optimization ability of the GA-SVM method is presented. The experimental results of the GA-SVM, SVM and softmax classifiers based on the BRPN with only EPN improvement (BRPN-EPN) are shown. These experiments are performed on the PASCAL VOC 2007 test set. The BRPN-EPN model achieves a mAP of 77.2% with the softmax classifier (Table 11). Moreover, the BRPN-EPN with the SVM classifier achieves a mAP of 76.9%. We can see that this value is 0.3 percentage points lower than that of the BRPN with the softmax classifier. The parameters *C* and *γ* are the key factors of the RBF kernel of the SVM. In general, the grid search method is used to find the suitable values of *C* and *γ*. However, this optimization method usually cannot find the global optimization values. Therefore, the classification ability of the SVM cannot meet our requirements. The GA method is applied to optimize *C* and *γ* to improve the classification performance of the SVM. We find that the mAP of the BRPN-EPN with GA-SVM classifier is 78.2%, which is the best in Table 11. Therefore, our proposed GA-SVM is effective.

**Table 11:** Comparison between the softmax, SVM and GA-SVM classifiers.

Classifier	mAP	aero	bike	bird	boat	bottle	bus	car	cat	chair	cow	table	dog	horse	motorbike	person	plant	sheep	sofa	train	tv
Softmax	77.2	77.9	82.4	77.9	67.7	67.9	85.8	84.9	86	59.8	82.4	73.2	85.7	84.7	84.1	76.8	50.2	76.8	74.3	83.9	80.9
SVM	76.9	77.2	82.3	77.2	67.3	67.4	84.2	84.7	86.6	60.3	82.4	73.3	85.1	84.7	83.4	76.7	50.1	75.6	74.7	84.5	80.6
GA-SVM	**78.2**	**78.4**	**83.4**	**78.4**	**68.9**	**68.4**	**86.1**	**86.1**	**87.8**	**61.7**	**83.4**	**74.4**	**86.3**	**85.9**	**84.9**	**78.2**	**51.3**	**77.1**	**76.2**	**85.7**	**82**

The computational complexity of the SVM classifier is O(*n*^3^). Additionally, the calculation complexity of the softmax classifier is O(*n*). Therefore, the computation time of the SVM classifier is greater than that of the softmax classifier. The average computational time of the Faster R-CNN model with the softmax classifier is approximately 0.5 seconds. However, the computational time of the Faster R-CNN model with the SVM classifier is approximately 200 seconds. Nevertheless, the choice of the classifier mainly affects the training time. The test time for our improved method is not significantly affected. Therefore, our method can be applied to real-time classification applications. Based on the experimental results, we can see that the classification capability of the BRPN model with the GA-SVM classifier is strengthened.

### Experiments on the KITTI dataset

The BRPN-GE, BRPN and other compared algorithms are tested on the KITTI autonomous driving dataset in this section. The KITTI dataset includes approximately 7481 training images and 7518 test images. Additionally, the KITTI dataset includes three object categories: cars, pedestrians and cyclists. The images are sampled from autonomous driving vehicles. Furthermore, easy, moderate and hard evaluation levels are provided in this dataset. The moderate level is used in general. Specifically, the pedestrian and cyclist data need 50% overlap. In addition, the car data requires 70% overlap. The AP value is applied to measure the object detection method capability. Table 12 shows the experiment results.

**Table 12:** Detection results on the KITTI dataset.

Method	Cars			Pedestrians			Cyclists		
	Easy	Moderate	Hard	Easy	Moderate	Hard	Easy	Moderate	Hard
FRCNN+Or	89.87	78.95	68.97	71.18	56.78	52.86	70.05	57.37	51.00
Mono3D	90.27	87.86	78.09	77.30	66.66	63.44	75.22	63.85	58.96
Faster R-CNN	87.9	79.11	70.19	78.35	65.91	61.19	71.41	62.81	55.44
Regionlets	86.50	76.56	59.82	72.96	61.16	55.22	70.09	58.69	51.81
DPM-VOC+VP	80.45	66.25	49.86	59.60	44.86	40.37	43.65	31.16	28.29
Occlusion Detector	79.2	56.8	38.5	56.4	42.5	35.9	61.3	47.3	36.4
PBLS_SRPN	89.7	88.6	78.6	82.4	70.5	65.1	78.72	70.3	61.6
BRPN-GE	89.1	88.5	80.7	81.8	70.2	64.2	77.9	69.8	61.2
BRPN	**90.4**	**89.9**	**81.2**	**82.7**	**71.3**	**65.5**	**78.8**	**70.4**	**61.9**

The best result is highlighted in bold.

The moderate category values are the most representative test results. From Table 12, we can see that the moderate category values for our proposed BRPN-GE model are better than those of the FRCNN+Or, Mono3D, Faster R-CNN, regionlets, Occlusion Detector and DPM-VOC+VP models on the car, pedestrian and cyclist data. The output features of the BRPN-GE model contain lower-level and higher-level features. In addition, different pooling sizes are added to the BRPN-GE. Therefore, the quality of the region proposals is strengthened. Specifically, the classifier is enhanced by introducing the GA-SVM classifier. Moreover, the classification and regression loss function is improved. In the BRPN, the GWO is applied to optimize the coefficients of the novel loss function. From the results, we can see that the moderate category values for the BRPN are the best. In other words, the improvements in the BRPN are effective.

### Runtime analysis

From Table 13, we can see that the detection speed of the BRPN is faster than that of the other methods. The reason is that fewer region proposals are generated by the BRPN. Based on a single NVIDIA Titan X GPU, our BRPN-based Faster R-CNN system has a frame rate of 5.2 fps for VGG-16. Consequently, our BRPN model achieves an outstanding detection speed.

**Table 13:** Detection frame rate of the different methods on the PASCAL VOC 2007 test set.

Approach	Fast R-CNN	Faster R-CNN	ION	MR-CNN	HyperNet	PBLS_SRPN	BRPN
Frame rate (fps)	0.5	5	1.25	2	5	5	5.2

## Conclusion

The experimental results of the BRPN-GE model are better than those of the Faster R-CNN model. The reason is that the feature sampling ability is improved by the EPN and the classification capability is enhanced by the GA-SVM classifier. Moreover, the best test results are achieved by the BRPN model. The classification loss function of the BRPN is improved when training on the negative samples. Additionally, more parameters are added to the regression loss function of the BRPN. Furthermore, the GWO is applied to optimize the parameters of the improved loss function. In other words, our proposed BRPN is effective for object detection in UGVs. However, processing steps are increased for introducing the optimization method in the training phase. Therefore the training time is affected compared to the original method. To solve this drawback, we will focus on improving the optimization technique to reduce the training time.

## Supporting information

S1 File Datasets description. The details of experimental datasets are described in the separated S1 File.
(DOCX)
