# Supplementary material for: A comprehensive swarming intelligent method for optimizing deep learning-based object detection by unmanned ground vehicles
Source: PLoS One. 2021 May 13;16(5):e0251339. doi: 10.1371/journal.pone.0251339 (PMC8118342; doi:10.1371/journal.pone.0251339)
Supplement: S1 File — The details of experimental datasets are described in the separated S1 File. (DOCX) [file pone.0251339.s001.docx]

Dataset Description

We appreciate your suggestion.

The PASCAL VOC 2007, 2012 [1] and KITTI [2] datasets are applied to train and test our proposed method. In S1 Table, the details of the datasets are shown as follow:

**S1 Table. Dataset information**

| No. | Dataset | No. of categories | No. of annotated objects |
| --- | --- | --- | --- |
| 1 | PASCAL VOC 2007 | 20 | 24,640 |
| 2 | PASCAL VOC 2012 | 20 | 27,450 |
| 3 | KITTI | 3 | 80,256 |

**PASCAL VOC 2007 and 2012 datasets**

PASCAL VOC 2007 and 2012 datasets are provided from the PASCAL Visual Object Classes (VOC) challenges. They are one of the most famous standardized image datasets for object detection. Both of them have 20 classes, where are person, bird, cat, cow, dog, horse, sheep, aeroplane, bicycle, boat, bus, car, motorbike, train, bottle, chair, dining table, potted plant, sofa, tv/monitor, respectively. In PASCAL VOC 2007, there are 9,963 images, containing 24,640 annotated objects. While there are 11,530 images, containing 27,450 annotated objects in PASCAL VOC 2012. Note that multiple objects from multiple classes may be present in the same image. Both of them have been split into 50% for training/validation and 50% for testing. The distributions of images and objects by class are approximately equal across the training/validation and test sets. Some example images are shown in S1 Fig. The datasets could be downloaded from online publicly as follow.

PASCAL VOC 2007 train/validation dataset:

http://host.robots.ox.ac.uk/pascal/VOC/voc2007/VOCtrainval_06-Nov-2007.tar

PASCAL VOC 2007 test dataset:

http://host.robots.ox.ac.uk/pascal/VOC/voc2007/VOCtest_06-Nov-2007.tar

PASCAL VOC 2012 train/validation dataset:

http://host.robots.ox.ac.uk/pascal/VOC/voc2012/VOCtrainval_11-May-2012.tar

PASCAL VOC 2012 test dataset:

<http://host.robots.ox.ac.uk:8080/eval/downloads/VOC2012test.tar>


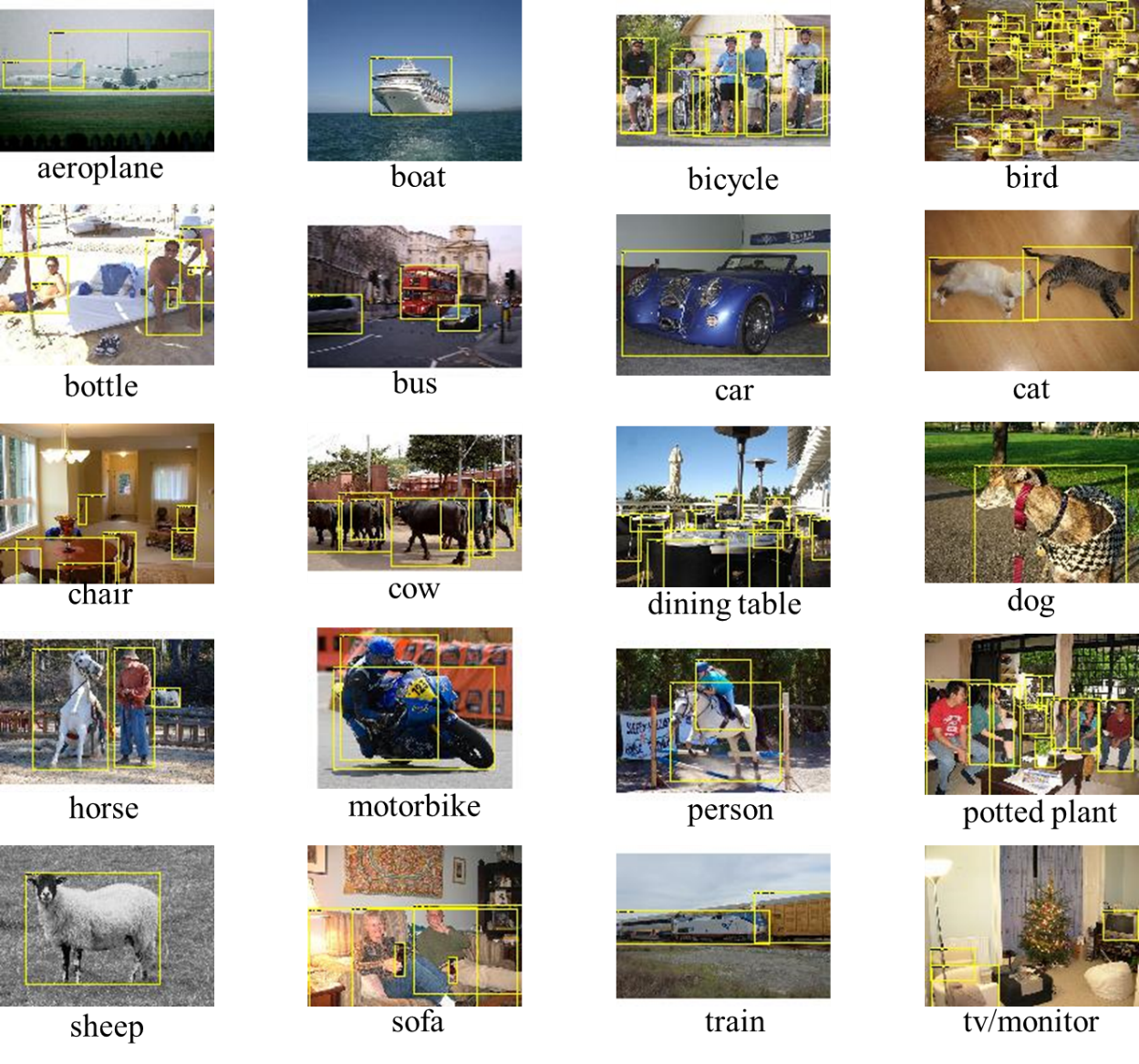


**S1 Fig.** Some example images of PASCAL VOC

**KITTI dataset**

KITTI dataset is a part of the KITTI Vision Benchmark Suite, which aim to develop novel challenging real-world computer vision benchmarks. It is famous in auto-driving. There are only 3 annotated classes in this dataset, where are car, pedestrian and cyclist, which are the most interested objects in the driving environment. The object detection benchmark consists of 7481 training images and 7518 test images, comprising a total of 80256 labeled objects. All images are color and saved as png. Some example images are shown in S2 Fig. The datasets could be downloaded from online publicly as follow.

KITTI dataset:

http://www.cvlibs.net/datasets/kitti/eval_object.php?obj_benchmark=2d

**
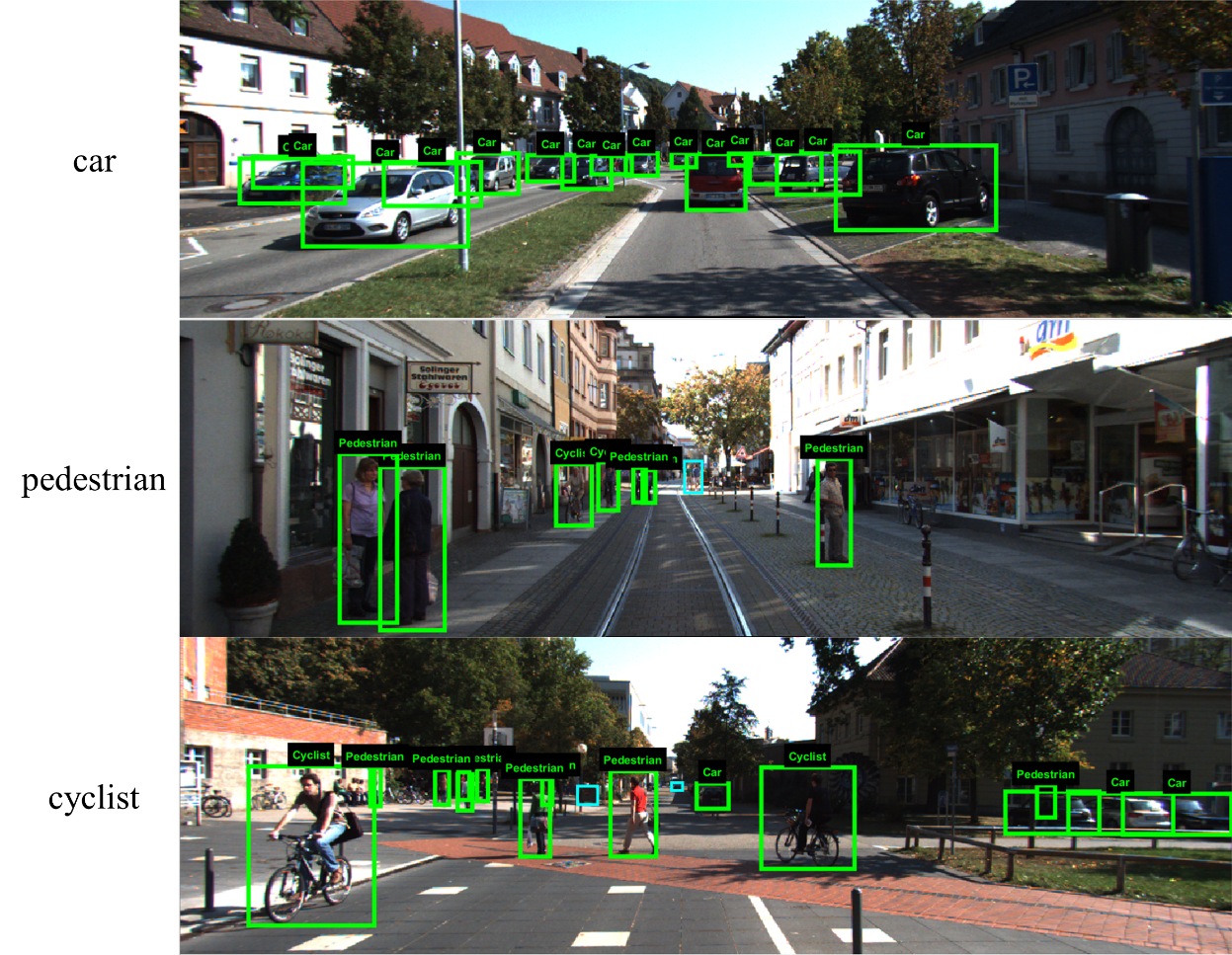
**

**S2 Fig.** Some example images of KITTI

**Reference**

1. M. Everingham, S. M. A. Eslami, L. Van Gool, C. K. I. Williams, J. Winn, and A. Zisserman, “The pascal visual object classes challenge: A retrospective,” Int. J. Comput. Vis., Jan. 2015, vol. 111, no. 1, pp. 98-136.
2. A. Geiger, P.Lenz, and R. Urtasun, “Are we ready for autonomous driving? the kitti vision benchmark suite,” IEEE Conference on Computer Vision & Pattern Recognition, 2012, 1, 6, 7.
